# First record of entomopathogenic nematodes from Yucatán State, México and their infectivity capacity against *Aedes aegypti*

**DOI:** 10.7717/peerj.11633

**Published:** 2021-07-02

**Authors:** Mariana B. Ávila-López, José Q. García-Maldonado, Héctor Estrada-Medina, David I. Hernández-Mena, Daniel Cerqueda-García, Víctor M. Vidal-Martínez

**Affiliations:** 1 Aquatic Pathology Laboratory. Centro de Investigación y de Estudios Avanzados del Instituto Politécnico Nacional Unidad Mérida, Carretera Antigua a Progreso,, Mérida, Yucatán, México; 2 Facultad de Medicina Veterinaria y Zootecnia, Departamento de Manejo y Conservación de Recursos Naturales Tropicales, Campus de Ciencias Biológicas y Agropecuarias, Universidad Autónoma de Yucatán, Mérida, Yucatán, México

**Keywords:** *Aedes aegypti*, Biological control, *Galleria mellonella*, *Heterorhabditis indica*, *Photorhabdus*, 16S rRNA

## Abstract

**Background:**

Biological control using entomopathogenic nematodes (EPN) has demonstrated good potential to contribute to the integral control of mosquito larvae, which as adults are vectors of diseases such as Dengue fever, Zika and Chikungunya. However, until now there are no records of the presence of EPN or their killing capacity in Yucatán state, southern México. The objectives of the current study were: (1) to report the entomopathogenic nematodes present in Yucatán soils and (2) to determine the killing capacity of the most frequent and abundant EPN against *Aedes aegypti* mosquito larvae and the microbial community developed by *Ae. Aegypti* exposed to this EPN.

**Methods:**

The nematodes were collected by the insect trap technique using the great wax moth *Galleria mellonella*. Internal transcribed spacer (ITS), 28S gene of ribosomal DNA and phylogenetic analyses were performed to identify the EPN. For the bioassay, four concentrations of the most frequent and abundant EPN were tested: 1,260:1 infective juveniles (IJs) per mosquito larvae, 2,520 IJs:1, 3,780 IJs:1 and 5,040 IJs:1. High-throughput sequencing of the 16S rRNA gene was used to identify bacterial amplicon sequences in the mosquito larvae infected with EPN.

**Results:**

Six isolates of *Heterorhabditis* were recovered from 144 soil samples. *Heterorhabditis indica* (four isolates) was the most frequent and abundant EPN, followed by *Heterorhabditis* n. sp. (two isolates). Both nematodes are reported for the first time for Yucatán state, Mexico. The concentration of 2,520 IJs:1 produced 80% of mosquito larvae mortality in 48 h. Representative members of *Photorhabdus* genus were numerically dominant (74%) in mosquito larvae infected by *H. indica*. It is most likely that these bacteria produce secondary toxic metabolites that enhance the mortality of these mosquito larvae.

## Introduction

Biological control using entomopathogenic nematodes (EPN) has demonstrated good potential to contribute to the integral control of pests of agricultural and medical importance (Chitra, Sujatha & Alagarmalai, 2017). In the case of nematodes of the families Steinernematidae and Heterorhabditidae, their killing capacity is based on their symbiotic relationship with bacteria of the genera *Xenorhabdus* and *Photorhabdus*, as well as on their capacity to infect insect hosts. During the EPN life cycle, the infective juvenile (IJs) enters the host’s body through natural openings such as mouth, anus and spiracles. *Steinernema* can even penetrate the insect’s integument (Peters, 1994; Peters & Ehlers, 1997). In the case of *Heterorhabditis*, this is done with the help of a cuticule projection resembling a tooth. Some authors even speak about a dorsal tooth (e.g., Nguyen et al., 2004). Once inside the host, the IJs migrate to the hemocoel where they release their symbiont bacteria (Bedding, Akhurst & Kaya, 1993). The bacterial community produces secondary metabolites with cytotoxic, antimicrobial, antiparasitic and insecticide activity, causing septicaemia in a period of 24 to 48 h after infection (Bode, 2009). These nematodes feed on the host tissues and reproduce for two or three generations, depending on the availability of food resources and the size of the host (San-Blas, Gowen & Pembroke, 2008). Then, the free-living stage IJs migrate, looking for another insect host to parasitise (Akhurst, 1993).

The monoxenic relationships between *Steinernema*-*Xenorhabdus* and *Heterorhabditis*-*Photorhabdus* have been frequently tested (Eivazian, Mohammadi & Girling, 2017), although bacteria such as *Providencia* sp., *Ochrobactrum* sp., *Pseudomonas* sp., and *Alcaligenes faecalis* have also been isolated from EPNs, without a clear understanding of their role in such interactions (Jackson et al., 1995; Babic et al., 2000; Gouge & Snyder, 2006). In insects, most microbiota studies have been based on the intestinal bacterial or haemolymph composition of infected insects (Engel & Moran, 2013). Indeed, diaxenic associations of non-canonical bacteria such as *Pseudomonas aeruginosa* have been found in the IJs of different generations of *H. indica*, favouring the infection of hosts such as *Galleria mellonella*, *Tenebrio molitor*, *Heliothis subflexa*, and *Diatraea magnifactella* when were combined with *Photorhabdus luminescens* (Salgado-Morales et al., 2019).

During the last few years, several studies have focused on the isolation of native EPN as biological control tools to fight against mosquitoes that may transmit important human diseases, such as dengue, Zika, Chikungunya and yellow fever (Kovendan et al., 2018). Recently, Dilipkumar et al. (2019) isolated *Steinernema siamkayai*, *Heterorhabditis indica*, *Steinernema glaseri* and *Steinernema abbasi* and evaluated their biocontrol potential against *Aedes aegypti*, *Anopheles stephensi* and *Culex quinquefasciatus* larvae. In the same way, in Thailand, Yooyangket et al. (2018) evaluated larvicidal activity of symbiotic bacteria belonging to EPN against *Ae. aegypti* and *Ae. albopictus* larvae. In Mexico, there are very few reports of native EPNs. For example, Girón et al. (2012) isolated *Heterorhabditis mexicana*, *Steinernema carpocapsae* and *Steinernema feltiae* from of Oaxaca soils. In Sonora, Stock, Rivera-Orduño & Flores-Lara (2009) isolated *Heterorhabditis sonorensis* (currently, *Heterorhabditis taysearae*; Dhakal et al., 2020) in nymph corpses of *Diceroprocta ornea* (Homoptera: Cicadidiae) in asparagus crops. Most of the EPN isolated in Mexico have been tested for the biological control of pests of agricultural importance (Stock, Rivera-Orduño & Flores-Lara, 2009; Salgado-Morales et al., 2019).

Mexico also has a very important problem with the insidious capacity of *Ae. aegypti* for transmitting viral diseases such as dengue fever, with 268,458 cases in 2019 (https://www.paho.org/data/index.php/en/mnu-topics/indicadores-dengue-en/dengue-nacional-en/252-dengue-pais-ano-en.html?). The official actions to control the vector spread have been the elimination of breeding grounds and the use organophosphate and organochlorine pesticides. However, an important drawback of these compounds is that they cause alterations to the nervous, immunological, respiratory, endocrine and reproductive systems of humans, not to mention resistance and persistence related to the environment (Blair et al., 2014). In rural and urban zones, the use of such pesticides represents the main cause of groundwater pollution (Olvera et al., 2008). Polanco et al. (2015) reported on the presence of organochlorine pesticides like DDT at a concentration of 16 ppb in the aquifer of the Yucatán state, Cenotes Ring. Because of this, it is extremely important to look for other environmentally friendly alternatives to control the *Ae. aegypti* vector life cycle and one of these alternatives are the EPNs.

Our quest for EPN in the Yucatán revealed the presence of two species. Since EPNs are pathogenic to insects, we hypothesise that the most frequent and abundant species (*H. indica*) should be able to produce high mortality (>70%) in *Ae. aegypti* mosquito larvae. Under this context, the purpose of the current study was two-fold: (1) to report for the first time the presence of two species of entomopathogenic nematodes from Yucatán state soils, and (2) to determine the killing capacity and microbial community present in *H. indica* against *Ae. aegypti* mosquito larvae.

## Materials & methods

**Figure 1:**
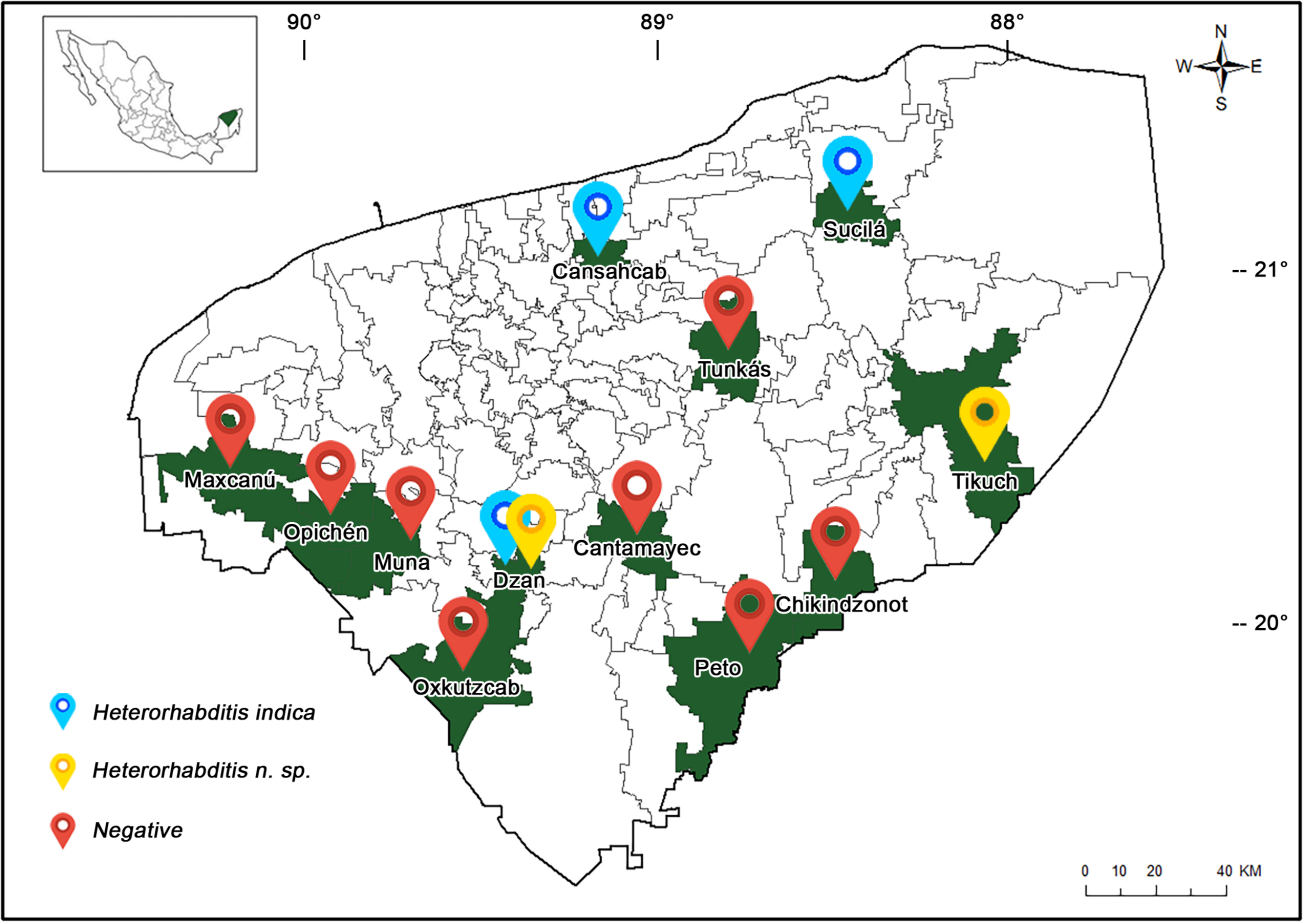
Map of Yucatán state showing the sites sampled for entomopathogenic nematodes. Sampling sites marked with red denote absence of EPNs, while sampling sites marked with blue and yellow were positive for EPNs.

During September and October 2018 (rainy season), soil samples were collected from 12 different locations of Yucatán state appertaining to the municipalities of Peto, Cantamayec, Tikuch, Cansahcab, Dzan, Sucila, Maxcanu, Opichen, Muna, Oxkutzcab, Tunkas and Chikindzonot (Fig. 1). Soil samples were collected from different cultivations of sour orange (*Citrus aurantium*) and sweet orange (*Citrus sinensis*). To obtain the EPN, four composite soil samples per location were used; these samples were placed in plastic containers (1 kg per flask) for a total of 144 samples. Each flask was punched in the bottom and before taking the sample from each flask, 15 cm were excavated using a small blade (Thanwisai et al., 2012). At the end of the collection, the soil samples were placed in foam coolers for transportation to the Aquatic Pathology Laboratory of the Centre for Research and Advanced Studies of the National Polytechnic Institute (Mérida Unit). *Galleria mellonella* cultured in the laboratory were used as insect traps for the EPN isolation (Bedding & Akhurst, 1975). Four *G. mellonella* larvae per flask were used and placed at room temperature for five days. The infected *G. mellonella* were placed in wet chambers until the IJ emergence, following the procedure suggested by Kaya & Stock (1997).

### Soil analysis

A soil composite sample (four subsamples of 250 g per site) was separated, weighed and labelled for physico-chemical analysis. The samples were processed at the Soil, Plant and Water Analysis Laboratory at the Universidad Autónoma de Yucatán (UADY), following a standard measurement procedure: particle size with densimeter (Gee & Bauder, 1986); redox and pH through a potentiometer (Patrick, Gambrell & Faulkner, 1996; Thomas, 1996); phosphorous by the Olsen method (Kuo, 1996); organic matter by colorimetric determination (Nelson & Sommers, 1996) and electric conductivity through potentiometry (Rhoades, 1996). Student’s *t*-test was used to determinate differences of physico-chemical characteristics between EPN positive and EPN negative soil samples (*p* ≤ 0.05).

### Polymerase chain reaction and sequencing of EPNs

**Figure 2:**
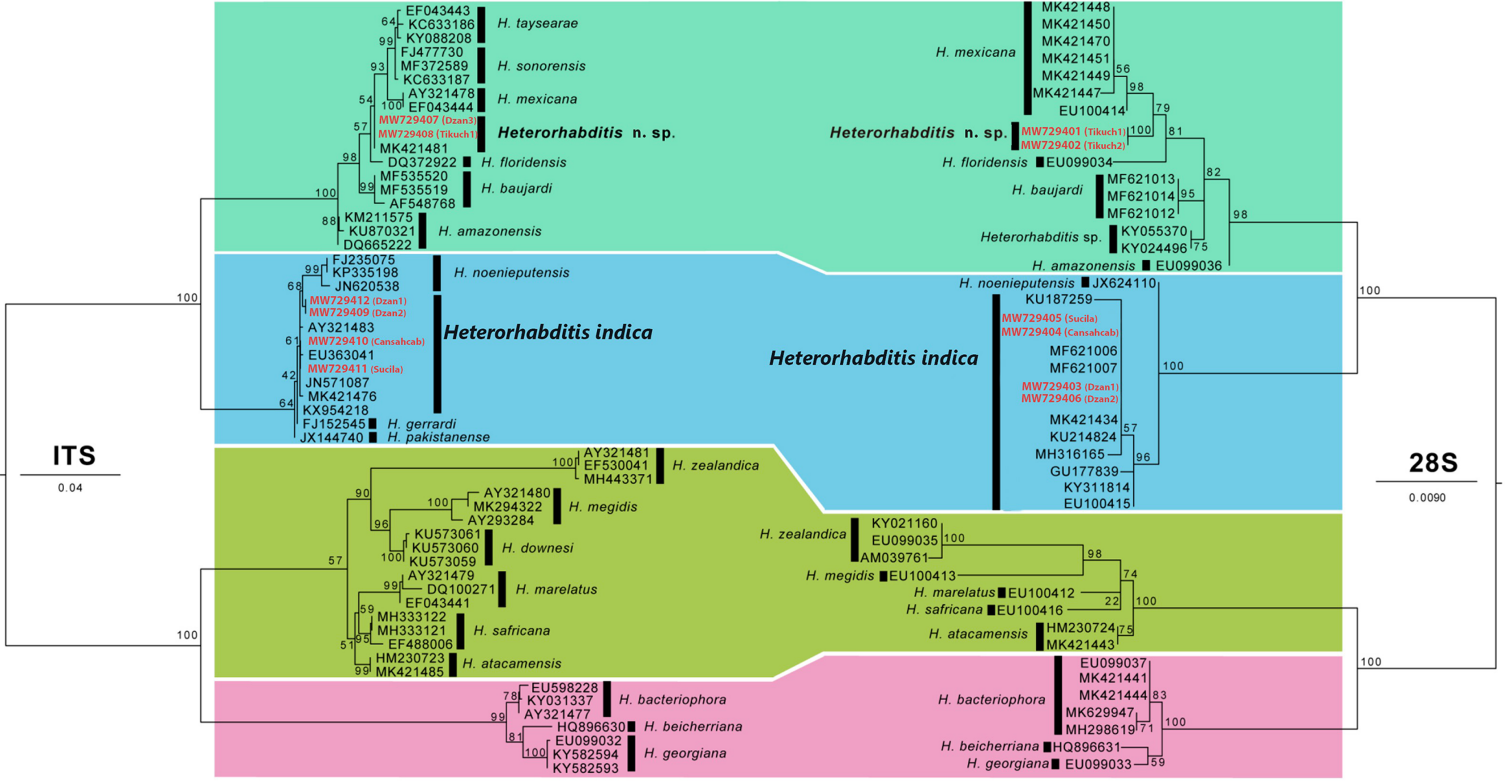
Phylogenetic relationships of species of the genus *Heterorhabditis* using the maximum likelihood method to generate the phylogenetic trees. The tree obtained with ITS is shown on the left side and the resulting tree with 28S is shown on the right side. The newly generated sequences are highlighted in red. Numbers near the tree nodes represent the bootstrap support values and the scale bar indicates the number of substitutions per site.

For DNA extraction, two adult nematodes were cut into small pieces using a sterile scalpel to reduce cell lysis time according to the protocol of the DNeasy Blood and Tissue kit (Qiagen™). The ITS region and the 28S gene of ribosomal DNA were amplified by the polymerase chain reaction (PCR). For the PCR mix in each tube we added the follow: 12.5 µl of Green GoTaq Master Mix (Promega, Madison, WI, USA), one µl of each primer (10 µm), 8.5 µl of distilled water and two µl of genomic DNA for a final volume of 25 µl. The primers used in this study for ITS were the forward TW81 5-GTTTCCGTAGGTGAACCTGC-3, plus the reverse AB28 5-ATATGCTTAAGTTCAGCGGGT-3 (Nthenga et al., 2014). For the 28S rRNA gene, the primers used were 391F 5′-AGCGGAGGAAAAGAAACTAA-3′ (Nadler & Hudspeth, 1998) and 536R 5′-CAGCTATCCTGAGGGAAAC-3′ (García-Varela & Nadler, 2005). All PCR reactions were run in an Axygen^®^ MaxyGene™ II thermocycler. For ITS, the following amplification conditions were used: one cycle of pre-denaturation at 94 °C for 5 min, followed by 35 cycles of denaturation at 92 °C for 30 s, annealing at 47 °C for 45 s, extension at 72 °C for 90 s and finally an extension at 72 °C for 10 min. Subsequently, the following amplification conditions were used for the 28S PCR: one cycle of pre-denaturation at 94 °C for 5 min, followed by 35 cycles of denaturation at 94 °C, annealing at 50 °C, and extension at 72 °C for 1 min each temperature and, then a final extension at 72 °C for 10 min. PCR products were verified by electrophoresis on a 1% agarose gel using 1X TAE buffer at 90 V for 45 min in a BioRad Sub-Cell^®^GT agarose gel electrophoresis system using a Promega^®^ DNA leader of 1 KB molecular weight as a reference. The PCR products were visualised in a BioDoc-It^®^ Imager. PCR products were commercially sequenced by GENEWIZ (South Plainfield, NJ, USA). Subsequently, the consensus sequences of ITS and 28S were aligned with the sequences obtained for each primer using Geneious Pro 4.8.4^®^ (Biomatters Ltd., Auckland, New Zealand). The consensus sequences of each nematode were deposited in the GenBank database with accession numbers MW729401 to MW729412 (Fig. 2).

### Phylogenetic analysis of EPNs

For the identification of the EPN that in both cases belonged to the genus *Heterorhabditis*, the consensus sequences of ITS and 28S rRNA gene of our specimens were subjected to BLAST (Altschul et al., 1990) alignment against the nucleotide database at National Centre for Biotechnology Information (NCBI). Subsequently, the sequences generated in this study were aligned with sequences available in GenBank belonging to other species of *Heterorhabditis*. Two datasets were generated, one for ITS and another for 28S. *Heterorhabditidoides* was used as outgroup. The alignment of each data set was performed with ClustalW (Thompson, Higgins & Gibson, 1994), implemented in the website http://www.genome.jp/tools/clustalw/, with the approach ‘SLOW/ACCURATE’ and weight matrix ‘CLUSTALW (FOR DNA)’. The extremes of the alignment were trimmed to match the length of our sequences. The nucleotide substitution model was estimated with the program jModelTest v2 (Darriba et al., 2012). Phylogenetic analysis was run under Maximum Likelihood (ML) with RAxML v. 7.0.4 (Stamatakis, 2006). A first phylogenetic analysis was performed with Raxml to discard sequences of GenBank that may have problems of taxonomic identification in each data set. Then, a second phylogenetic analysis was performed with 10 replicates and 1,000 bootstrap repetitions to obtain the best phylogenetic hypothesis of the ITS and 28S datasets. The ML trees were visualised in FigTree v.1.4.3. (Rambaut, 2016). Molecular variation of 28S data sets was estimated using uncorrected p distances (p-distances) with the software MEGA v.6 (Tamura et al., 2013).

### Experimental infection

For the assays we used the most frequent and abundant entomopathogenic nematode species recovered from the *G. mellonella* infections, i.e., *H. indica*. We used flasks with a capacity of 1.9 l with 400 ml of bubbled tap water, and the following procedure. Four concentrations of nematodes were used: 1:1,260 mosquito larvae/infective juveniles (l/[IJs]), 1: 2,520 l/IJs, 1: 3,780 l/IJs, 1: 5,040 l/IJs and a control per concentration (mosquito larvae without nematodes) with each concentration replicated four times. Each treatment and control flask contained ten *Ae. aegypti* larvae in the fourth stage. The experimental infection was replicated two times, with very similar results (Fig. S1 presents mosquito larvae survival in the first experiment). Consequently, we only present the outcomes of the last experimental infection. The *Ae. aegypti* eggs were raised in the Laboratory of Aquatic Pathology of the Centre for Research and Advanced Studies of the National Polytechnic Institute from eggs kindly donated by Centre for Research Dr. Hideyo Noguchi. The mosquito larvae mortalities were monitored every 4 h until 48 h post-exposure (time allowed before the transformation of larvae into pupae). Dead mosquito larvae (those without movement when touched with a fine painting brush) were washed (three times) with distilled water and fixed in DNA/RNA Shield.

The survival of *Ae. aegypti* larvae was estimated through a survival analysis (Kaplan–Meier Estimate). This analysis is a nonparametric statistical method to estimate the probability of the mosquito larvae survival after infection with *H. indica* through time. To determine the dosage at which *H. indica* caused 50% of mortality in *Ae. aegypti* larvae, a probit analysis was conducted following Finney (1971) procedure. The software used for survival and probit analysis was MedCalc^®^ (MedCalc Software Ltd., Ostend, Belgium).

### Bacterial detection by 16S rRNA sequencing

In order to get a broad comparison of the bacterial composition from infected and non-infected *Ae. aegypti* larvae, genomic DNA was extracted from a pool of three larvae for each type of samples (infected and non-infected) with the DNAeasy blood and tissue extraction kit (Qiagen, Valencia, CA, USA). DNA quality was verified by agarose gel (1%) electrophoresis. PCR Amplification of hypervariable V3 and V4 regions of 16S rRNA, library preparation and Illumina sequencing were performed as previously described in Valencia-Agami et al. (2019). Bioinformatic processing were done with the QIIME2 (2019) pipeline (Caporaso et al., 2010). The error correction, denoising and ASVs taxonomical assignation were performed as previously reported in Cadena et al. (2019).

## Results

From the 12 collection sites in the Yucatán state, four sites were positive for EPNs (Cansahcab, Sucila, Ticuch and Dzan). From the 144 soil samples, six isolates were positive for Heterorhabditidae (4.16%). Most of the nematodes were isolated from Dzan, Yucatán. Based on the nucleotide sequence analysis of 28S and ITS, we concluded that four isolates belonged to the species *Heterorhabditis indica* and two isolates were identified as *Heterorhabditis* n. sp.

### Molecular identification and phylogenetic analysis of *Heterorhabditis*

The comparison of nucleotide sequences with the sequences published in GenBank through BLAST allowed us to molecularly identify our EPN from Yucatán state, where some of our specimens had a high percentage of similarity with *H. indica* (99.73–100% in ITS and 99.89–100% in 28S). With respect to the phylogenetic analysis, the final ITS data set consisted of 57 sequences representing 17 species, and the alignment had a length of 1,092 bp. The substitution model selected for this data set was GTR+I. Nucleotide frequencies were A = 0.254, C = 0.203, G = 0.255 and T = 0.288. The ML value of the tree was −ln = 4,451.710121. The phylogenetic tree obtained with the ITS data set showed that the new sequences of this study were grouped into two clades, one corresponding to *H. indica* and another one that belongs to an unidentified species: *Heterorhabditis* n. sp. (Fig. 2). In Dzan, Yucatán the two species *H. indica* and *Heterorhabditis* n. sp. were present, while only *Heterorhabditis* n. sp. was found in Tikuch. In the phylogenetic tree, *Heterorhabditis* n. sp. was phylogenetically closest to the clade formed by *Heterorhabditis taysearae* + *Heterorhabditis mexicana* (Fig. 2), with an interspecific genetic distance of 1.31% (10 nucleotides) with both species and there was no intraspecific variation between the specimens of *Heterorhabditis* n. sp. All sequences identified as *H. indica* were grouped with other sequenced *H. indica* specimens, in a clade that also contained to *H. noenieputensis* (Fig. 2). The genetic distance between *H. indica* and *H. noenieputensis* was 1 to 1.2% (7 to 9 nucleotides). The intraspecific genetic distance of *H. indica* was from 0 up to 0.4%. For the 28S gene, the data set consisted of 45 sequences representing 16 species, with a length of 934 bp. The substitution model selected was GTR+G+I. Nucleotide frequencies were A = 0.260, C = 0.193, G = 0.295 and T = 0.252. The ML value of the tree was −ln = 2,146.711150. The phylogenetic relationships of both *Heterorhabditis* species with the 28S gene were practically the same as those obtained with the ITS. As in ITS, the specimens sequenced in this study were grouped into two independent clades. The nematodes from Tikuch were separated as *Heterorhabditis* n. sp. and the genetic distance between the *Heterorhabditis* n. sp. and *H. mexicana* specimens was 0.5% to 0.7% (4 to 6 nucleotides) with no intraspecific differences between the *Heterorhabditis* n. sp. On the other hand, the nematodes from Cansahcab, Dzan and Sucila were grouped as *H. indica*, with an interspecific genetic distance of 0.3% (3 nucleotides) with *H. noenieputensis*. The intraspecific genetic distance of the *H. indica* was up to 0.2% (2 nucleotides).

### Soil analysis

*H. indica* and *Heterorhabditis* n. sp. were found in soils used for agriculture and cattle farming. The characteristics of the soil of the localities where these EPN were and were not found are presented in Table 1. Apparently, the relevant characteristics for the presence of these EPN in soil were chemical variables such as potassium and calcium (See Table 2). Significant differences in variables such as P (mg/kg), %OM and soil type were not found between EPN positive and EPN negative samples (*P* > 0.05).

**Table 1:** Mean of the measured soil parameters with the presence and absence of EPNs from Yucatán state.

Genus/species	Locality	Geographic coordinates	Soil type	Organic matter (%)	pH	Land use	Potassium (Cmol(+)/kg)	Calcium (Cmol(+)/kg)	Phosphorus (mg/kg)
*H. indica*	Cansahcab	21°9′16.02″ N 89°5′26.36″ O	Sandy clay loam	12.7	8.0	Cattle farm	1.5	53.8	133.6
*H. indica*	Sucila	21°8′58.98″ N 88°18′22.04″ O	Sandy clay loam	7.5	7.9	Cattle farm	2.3	24.6	89.9
*H. indica*/*Heterorhabditis* n. sp.	Dzan	20°22′36.1″ N 89°27′2.6″ O	Sandy clay loam	5.1	7.8	Field crop	14.7	80.2	1.6
*Heterorhabditis* n. sp.	Ticuch	20°42′5.82″ N 88°6′39.07″ O	Sandy loam	8.5	7.2	Cattle farm	0.3	17.0	0.02
Without EPNs	Maxcanu	20°35′34.31″ N 89°59′15.91″ O	Sandy loam	9.6	7.5	Industrial	2.5	43.3	58.6
Without EPNs	Opichen	20°33′17.34″ N 81°51′51.50″	Sandy loam	9.1	7.8	Forestry	3.0	46.0	801.1
Without EPNs	Muna	20°29′16.34″ N 89°43′36.18″ O	Sandy loam	12.4	7.4	Industrial	2.2	37.8	4.1
Without EPNs	Oxkutzcab	20°19′1.00″ N 89°25′52.79″ O	Clay	4.8	7.4	Citric crop	1.5	15.6	38.2
Without EPNs	Tunkas	20°54′5.45″ N 88°44′41.77″O	Sandy loam	9.5	7.8	Field crop	1.5	52.4	2430.5
Without EPNs	Chikindzonot	20°19′39.40″ N 88°29′23.66″ O	Clay	5.7	7.9	Forestry	2.3	30.2	0.3
Without EPNs	Peto	20°06′25.4″ N 88°52′45.6″ O	Clay	8.1	7.6	Forestry	1.7	33.4	2.2
Without EPNs	Cantamayec	20°28′38.40″ N 89°4′43.62″ O	Sandy loam	0.8	7.7	Field crop	0.5	26.1	1.3

**Table 2:** Mean difference between EPN positive and EPN negative soil samples parameters.

Parameter	Group 1	Group 2	N1	N2	Mean (1)	Mean (2)	Mean (1)–Mean (2)	T	*P* value
Phosphorus (mg/kg)	EPNs	Without EPNs	16	40	70.37	417.07	−346.70	−1.95	0.0575
Organic matter (%)	EPNs	Without EPNs	16	40	8.48	7.55	0.93	0.87	0.3906
Sand (%)	EPNs	Without EPNs	16	40	45.99	42.29	3.70	0.70	0.4889
Silt (%)	EPNs	Without EPNs	16	40	15.63	16.45	−0.82	−0.41	0.6830
Clay (%)	EPNs	Without EPNs	16	40	38.39	41.26	−2.88	−0.56	0.5752
Potassium (Cmol(+)/kg)	EPNs	Without EPNs	16	40	5.86	1.94	3.92	2.50	0.0247
pH	EPNs	Without EPNs	16	40	7.88	7.69	0.19	2.43	0.0183
Calcium (Cmol(+)/kg)	EPNs	Without EPNs	16	40	50.64	35.65	14.99	2.29	0.0337

### Experimental infection

**Figure 3:**
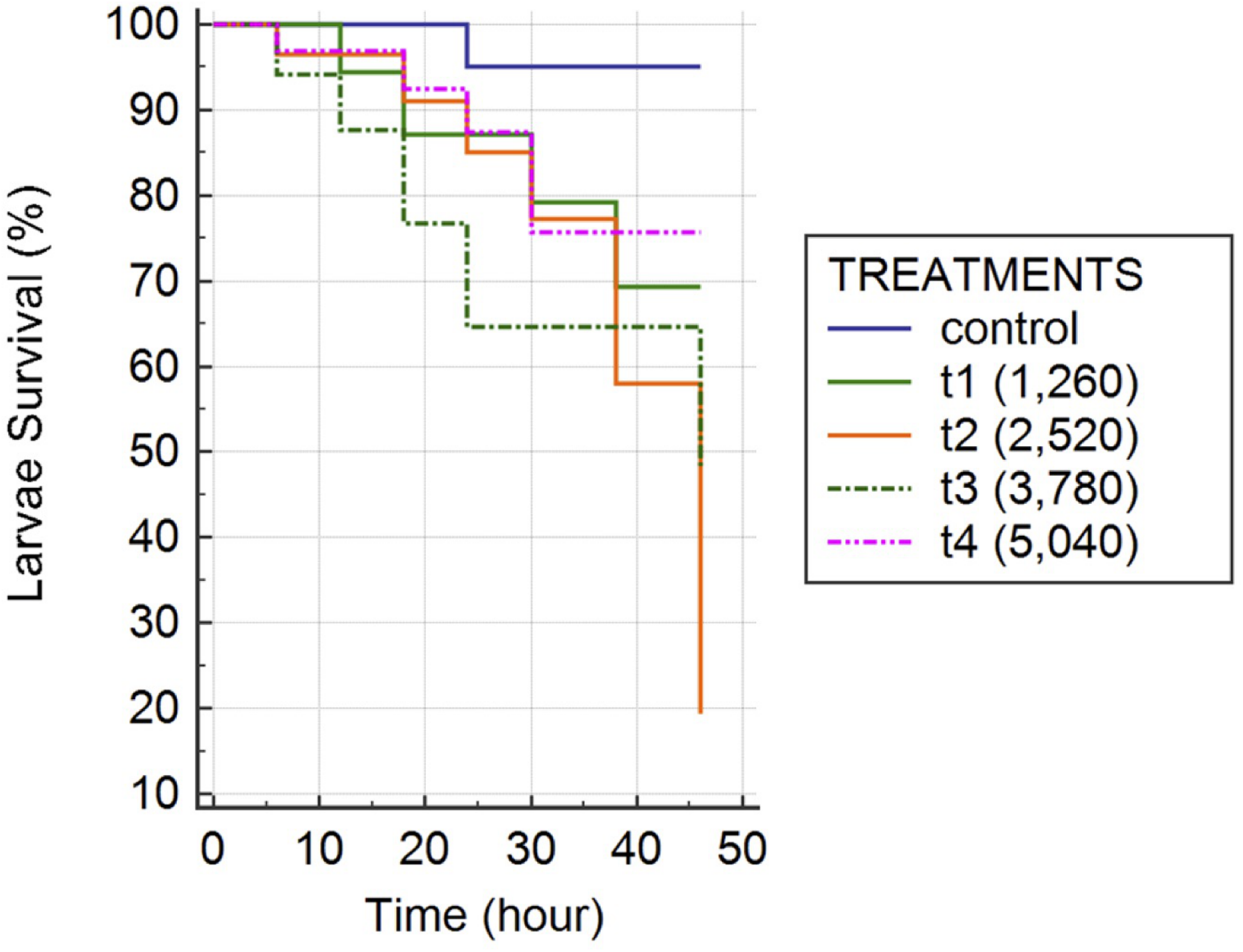
Kaplan-Meier overall survival curves comparing the mortality of *Aedes aegypti* larvae after exposure to infective juveniles of *Heterorhabditis indica*. Control contained no infective juveniles. The treatments units were the number of infective juveniles of *H. indica* per mosquito larvae. Each treatment had 10 mosquito larvae.

The percentage of survival values of *Ae. aegypti* mosquito larvae exposed to *H. indica* are shown in Fig. 3. The mean survival values of the mosquito larvae exposed to *H. indica* and their 95% confidence intervals are shown in Table 3. There was a significant difference in survival when comparing the average mortality of *Ae. aegypti* larvae between the various nematode concentrations and the control (Kaplan–Meier estimate, *P*-value = 0.0279). Figure 3 showed that the highest mortality (80%) in *Ae. aegypti* larvae was achieved using 1:2,520 l/IJs and 1:3,780 l/IJs after 46 h. The lowest mortality obtained was at 24% for the concentration with the highest number of nematodes (1:5,040 l/IJ). The lethal dose (LD50) was estimated at different exposure times (Table 4). The LD50 of *H. indica* at 24 hr was of 7,040 infective juveniles, with a confidence interval of 95% from 5,802 to 8,277 infective juveniles.

**Table 3:** Percentage survival values of *Aedes aegypti* mosquito larvae exposed to entomopathogenic nematodes *Heterorhabditis indica*.

Factor	Mean	SE	95% CI for the mean
Control (no nematodes)	44.900	1.072	[42.799–47.001]
T1 (1:1,260 l/IJs)	40.016	2.757	[34.612–45.420]
T2 (1: 2,520 l/IJs)	38.954	2.640	[33.780–44.128]
T3 (1: 3,780 l/IJs)	35.710	2.814	[30.195–41.225]
T4 (1: 5,040 l/IJs)	40.524	2.196	[36.219–44.829]

**Note:**

The acronyms were as follows: SE, standard error; 95% CI for the mean, 95% confidence intervals (CI) for control and treatments (T1–T4); l/IJs, mosquito larvae/infective juveniles.

**Table 4:** Fifty percent lethal dose values (LD50) for *H. indica* against mosquito larvae third stage at different exposure times.

Exposure time (h)	LD50 (IJs/mosquito larvae)	95% Confidence limits
12	14,997	[11.545–19.481]
24	7,040	[5.802–8.277]
48	6,324	[5.189–7.458]

### Bacterial composition of *Ae. aegypti* larvae

**Figure 4:**
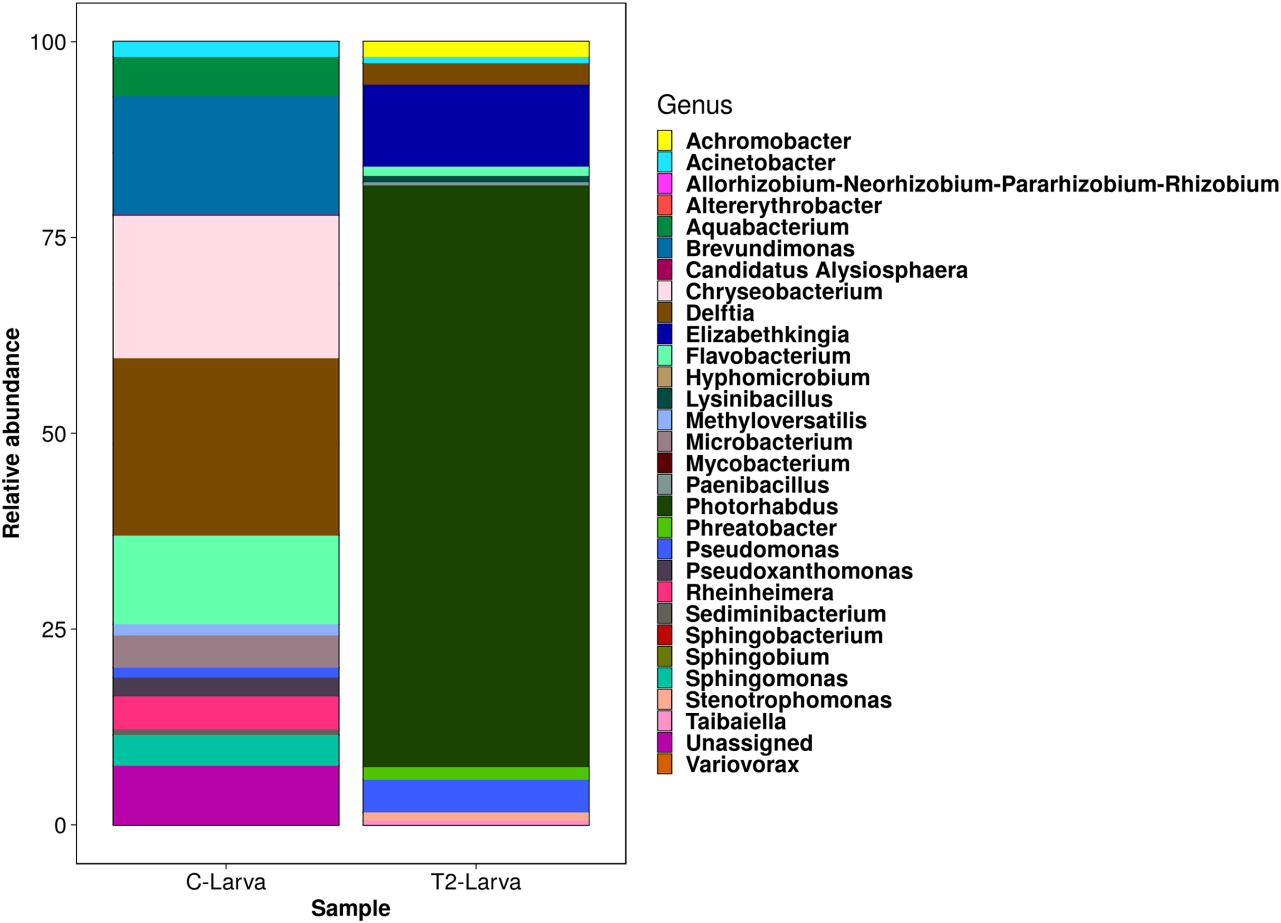
Relative abundance of bacteria in the larvae of *Aedes aegypti* infected with *Heterorhabditis indica*. The colours represent the bacterial diversity of the genera present in the mosquito larvae. Acronyms were as follows: C-Larva: mosquito larvae from the control treatment without nematodes; T2-Larva: mosquito larvae from treatment two (2,520 infective juveniles of *Heterorhabditis indica* per mosquito larva).

Bioinformatic analyses of the 16S rRNA sequences indicated that the pool of mosquito larvae infected by *H. indica* was composed by twenty ASVs corresponding to 9 families and 12 genera (Fig. 4). In which, at a lower taxonomic level, *Photorhabdus* was the dominant genus in the sample (74.2%). However, *Elizabethkingia* (10.4%), *Pseudomonas* (4.1%), *Delftia* (2.7%), *Achromobacter* (1.9%) *Phreatobacter* (1.6%) and other less abundant (<1%) genera were also detected (Fig. 4). In contrast, the pool of non- infected mosquito larvae (control), exhibited 19 ASVs from 13 families and 14 genera (Fig. 4). *Delftia* (22.61%), *Chryseobacterium* (18.19%), *Brevundimonas* (15.06%) and *Flavobacterium* (11.34%) were the most abundant genera (Fig. 4).

## Discussion

*Heterorhabditis indica* and *Heterorhabditis* n. sp., are reported for the first time in Yucatán state, México. The finding of these nematode species was corroborated by the ITS and 28S genes. Our original hypothesis that *H. indica* would be able to produce a high percentage of mortality (>70%) in *Ae. aegypti* mosquito larvae was supported. This was confirmed since *H. indica*, in association with its bacterial composition, was able to induce 80% mortality in *Ae. aegypti* larvae at the concentration of 2,520 IJs/I by 48 h post-infection. This is the first record of EPN present in Yucatán state soil, and the results obtained were promising in terms of the pathogenicity of this nematode and its potential as a biological control alternative.

### Molecular identification and phylogenetic analysis of *H. indica* and *Heterorhabditis* n. sp.

The analysis of the nucleotide sequences and phylogenetic analysis of the 28S and ITS of the isolated nematodes of Dzan, Cansahcab and Sucila in this study allowed us to corroborate the taxonomic identification of two species of *Heterorhabditis*. In the case of *Heterorhabditis* n. sp., the phylogenetic analysis and genetic distances indicated that this nematode is a genetically different species from those sequenced and previously described. The finding of *H. indica* in Yucatán state is not surprising since apparently is a species distributed worldwide (e.g., Saleh et al., 2001; Dolinski et al., 2008; Bruno et al., 2020). Phylogenetic analysis positioned *H. indica* from Yucatán state within the Indica group, named by Andaló, Nguyen & Moino (2006) (Nguyen, Shapiro & Mbata, 2008; Malan, Knoetze & Tiedt, 2014; Dhakal et al., 2020). All species of the Indica-group are found in the tropics and subtropics zones and this group is divided into two subclades: the Indica-subclade conformed of *H. indica* and *H. noenieputensis*, and the Baujardi-subclade with *H. baujardi*, *H. amazonensis*, *H. floridensis*, *H. mexicana* and *H. taysearae* (Dhakal et al., 2020). Particularly, the two EPN species of our study were phylogenetically associated with each of the previously described subclades. *Heterorhabditis* n. sp. from Dzan and Tikuch was grouped in the Baujardi-subclade, and there were no intraspecific differences between the specimens sequenced with both markers. The phylogenetic position of *Heterorhabditis* n. sp. makes sense geographically, because it was grouped with species that have been described in North America, such as *H. mexicana* and *H. floridensis* (Nguyen et al., 2004; Nguyen, Mrek & Webster, 2006).

Our study reports for the first time, the presence of *Heterorhabditis* n. sp. in the Yucatán state. It should also be noted that the sequences of the undescribed species are grouped with two sequences that were identified as *H. mexicana* in the GenBank; however, the phylogenetic analysis showed that this sequence does not correspond to the species described by Nguyen et al. (2004).

### Soil samples

The soil type (sand, silt, clay) did not seem to be as important as the physico-chemical composition of soil for the presence of EPN. The nematodes were present in soil with alkaline pH going from 7.2 to 8. It has been proved that the alkaline levels (10) or acid levels (5.6) reduce survival of EPN, in comparison to values closer to neutrality (Plante et al., 2006). This result coincides with those reported by Rodríguez et al. (2009) where it was observed that *Heterorhabditis* is present in soils with an alkaline pH compared to *Steinernema*.

The potassium concentration reported in soil samples in this study was 0.03 to 14.7 (Cmol(+)/kg) and 17 to 80.2 (Cmol(+)/kg) of calcium. Alumai et al. (2006) mentioned not finding an individual correlation between the presence of EPN and factors such as calcium, potassium and pH in turfgrass, but together contribute to the cation exchange capacity of the soil. Thus, apparently K and Ca could be the factors that influenced the presence and distribution of these EPNs in Yucatán State soil.

Despite of the fact that significant differences were not found between soil types, EPN were found in soils where agriculture and stockbreeding are carried out and were classified as sandy-clay soils. In addition, Dolinski et al. (2008) suggest that *H. indica*, together with other EPN typical of tropical regions, can be found in environments with a relative high percentage of sand.

Yooyangket et al. (2018) reported 13 isolates of *Heterorhabditis* and 14 isolates of the *Steinernema* in clay soils of the Nam Nao National Park, Thailand. Therefore, the difference between the Yucatán and Thai soils would be in the sand contents. Whether or not this sand content is acting as a limiting factor for the survival of the Yucatán EPN should be tested experimentally. In the same way, it is important to extend this research to new sampling points in the Yucatán State probably without the influence of agriculture and stockbreeding to determine whether the poor EPN species richness detected is a regional pattern. This is important because other authors have noted that edaphic factors may dramatically influence the presence of EPN (Lacey & Kaya, 2000).

### Experimental infection

Our results demonstrated that *Ae. aegypti* larvae were susceptible to *H. indica* (and their bacteria) recovered from Yucatán state at 2,520 nematodes per mosquito larvae (Fig. 3). It was expected that a higher number of nematodes would produce higher mortality. However, the 5,040:1 concentration reached only 25% mortality in mosquito larvae. A possible explanation for the poor performance of high concentrations of nematodes in producing mosquito larvae mortality is that of intraspecific competition by nutrients and space, which in turn reduced their infectivity and survival when the nematode concentration increases. This is a process that has been observed previously by Sen-Selvan, Campbell & Gaugler (1993), who compared the effect of increasing *S. carpocapsae* and *H. bacteriophora* in *G. mellonella* larvae. The authors reported that when the number of nematodes per host increased, the percentage of penetration decreased. Certainly, our results suggest that the use of nematode concentrations as high as 5,040 IJs/I is not effective nor economically feasible. However, now that the most effective nematode concentration to produce mosquito larvae mortality has been established (2,520 IJs/I), the following task will be to decrease the number of IJs mantaining a similar mortality level.

In terms of mosquito larvae mortality, Peschiutta, Cagnolo & Almirón (2014) reported the susceptibility of *Ae. aegypti* to *H. bacteriophora*, obtaining 84% mortality at a concentration of 750 IJs per mosquito larvae. Our results contrast with those reported in *H. bacteriophora*, since a lower concentration of nematodes was enough to obtain high mortality in *Ae. aegypti*. We consider the results obtained here as promising since the Yucatán State strain of *H. indica* produced a high mortality level in normal tropical environmental conditions with regards to temperature (>29.6 °C; >85.3 °F), CaCo_3_ (408 ppm) and pH (6.88) compared to those of Peschiutta, Cagnolo & Almirón (2014) developed in laboratory conditions at 26 °C. However, the percentage mortality reached with *H. indica* from Yucatán state was higher than that obtained by Pandii et al. (2008) who tested the infective capacity of *S. carpocapsae* and *H. indica* (Local Thai strain) in *Culex gelidus*, obtaining a mortality of 63% and 13%, respectively, at a concentration of 4,000 IJs per mosquito larvae. Clearly, additional bioassays exposing *Ae. aegypti* larvae to lower concentrations of *H. indica* from Yucatán state are necessary. Although in this work we did not evaluate the physiological responses of mosquito larvae to *H. indica* infection, it is known that EPN can cause mortality by suppressing the immune response of insects by reducing the number of haemocytes and phenoloxidase levels (Lalitha et al., 2018).

### Differences in the bacterial composition of infected and non-infected *Ae. aegypti* larvae

16S rRNA sequences allowed a broad comparison of the bacterial composition of infected and non-infected *Ae. aegypti* larvae. Several differences were observed in the two type of samples. However, the main difference was that *Photorhabdus* was the dominant genus in the infected mosquito larvae, representing 74.2% of the total microbial composition. Thus, we suggest that the endosymbiotic *Photorhabdus* most likely transported by *H. indica*, was able to infect *Ae. aegypti* larvae; something that did not happen in the non-infected mosquito larvae. Moreover, *Elizabethkingia* was also found as part of the microbial community of *Ae. aegypti* larvae infected by EPN. Boissière et al. (2012) reported *Elizabethkingia* spp. in 68% *Anopheles gambiae* mosquito collected in Cameroon. It is very likely that some species of *Elizabethkingia* are symbionts of mosquitoes, since they have been found through the life cycle of *Ae. aegypti* (Coon et al., 2014).

Only four genera were shared between the control and infected mosquito larvae: *Acinetobacter, Flavobacterium*, *Delftia* and *Pseudomonas*. Jiménez et al. (2016) reported species of *Pseudomonas* and *Delftia*, which are part of the EPN microbiota in species such as *Rhabditis regina*, with the capacity of causing mortality in *Phyllophaga* sp., *Anomalla* sp. and *G. mellonella*. Another bacterial genus found in the microbiota of mosquito larvae infected by *H. indica* was *Achromobacter* which, despite presenting a relative low abundance (1.9%), apparently could be also pathogenic to insects. For example, Poinar & Thomas (1966) isolated *Achromobacter nematophilus* from the nematode *Neoplectana* sp. while demonstrating its pathogenicity in *Galleria mellonella* larvae.

By high throughput sequencing, it was possible identify bacterial genera with low relative abundance (<1.2%) such as *Flavobacter, Stenotrophomonas, Acinetobacter, Lysinibacillus, Taibaiella* and *Paenibacillus*. Some of these bacteria have been reported to have low pathogenicity to mosquito larvae, especially those of the genera *Lysinibacillus* and *Paenibacillus* (Rojas-Pinzón & Dussán, 2017). Cambon et al. (2020) mentioned that the host’s ‘normal’ microbiota may be present in the host without causing damage, but under specific stress conditions such as infection with EPN, these opportunistic bacteria could manifest if the host immune system is suppressed. In the current study, the role these bacteria play during the infection process of the mosquito larvae or their interaction with the immune capacity of these larvae is unknown. For this reason, it would be pertinent to perform further experiments concerning the infections by *H. indica* bacterial symbionts and other bacteria considered as entomopathogenic, to formulate bacterial associations with greater pathogenic capacity. This is an interesting research avenue since it could provide us with new nematode-bacteria consortia acting on real environmental conditions against mosquito larvae.

In summary, our results suggest that the local strain of *H. indica* in association with their endosymbiotic bacteria have the infective capacity to induce 80% mortality in *Ae. aegypti* larvae. The high mortality of *Ae. aegypti* larvae apparently was related to bacteria of the genus *Photorhabdus*, since this was the numerically dominant endosymbiont compared to other non-symbiotic bacteria reported in this work. However, changes in the structure of the microbial community of *Ae. aegypti* larvae were observed as a result of exposure with *H. indica*. Even when EPN and/or their symbiotic bacterial associations appear as very promising biocontrol tools to kill mosquito larvae, it is important to be very careful about the release of these organisms into the environment, even if they are native. This is especially important for a region such as Yucatán state where native (and introduced) bees are important insects related to the pollination of wild plants, and beekeeping is an important economic activity in the countryside. Therefore, further studies should be performed to determine the susceptibility of beneficial insects such as European bees and stingless bees such as *Melipona* to EPN and the toxins released by their symbiotic bacteria.

## Supplemental Information

10.7717/peerj.11633/supp-1 Supplemental Information 1 Kaplan-Meier survival analysis. Kaplan-Meier overall survival curves comparing the mortality of *A. aegypti* larvae after exposure to infective juveniles of *H. indica*.

10.7717/peerj.11633/supp-2 Supplemental Information 2 28S *Heterorhabditis* sequences.

10.7717/peerj.11633/supp-3 Supplemental Information 3 ITS *Heterorhabditis* sequences.

## Additional Information and Declarations

### DNA Deposition

The following information was supplied regarding the deposition of DNA sequences: The sequences are available at GenBank: MW729401 to MW729412.

### Data Availability

The following information was supplied regarding data availability: Datasets on soil characteristics of the sampling sites where entomopathogenic nematodes were found and on the mosquito larvae mortality produced by entomopathogenic nematodes from Yucatán, Mexico are available at Zenodo: Mariana B. Ávila-López, Victor M. Vidal-Martínez. (2021). Dr. (researcher) (Version 1) [Data set]. Zenodo. DOI 10.5281/zenodo.4470024.
