# Supplementary figures and images for: First record of entomopathogenic nematodes from Yucatán State, México and their infectivity capacity against Aedes aegypti
Source: PeerJ. 2021 Jul 2;9:e11633. doi: 10.7717/peerj.11633 (PMC8256808; doi:10.7717/peerj.11633)

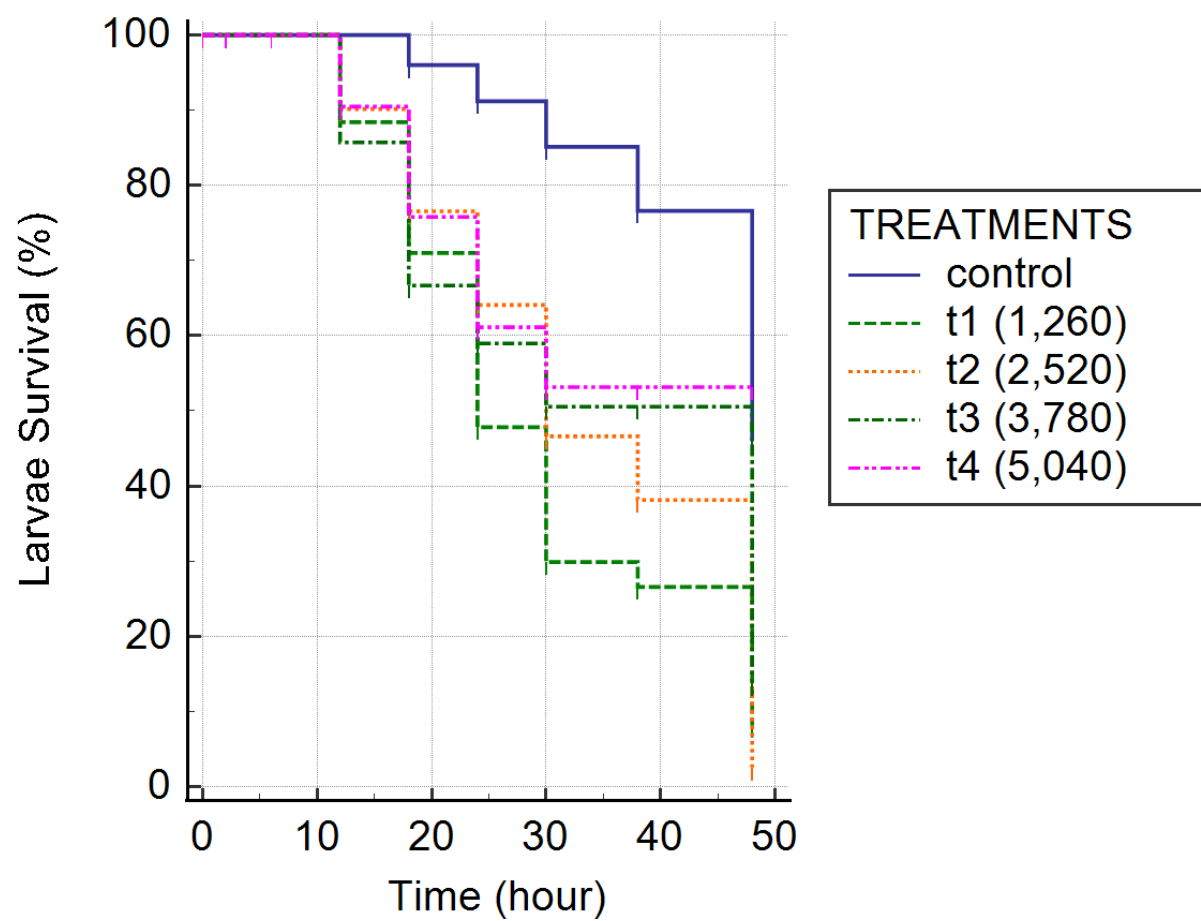

Supplement: Supplemental Information 1 — Kaplan-Meier overall survival curves comparing the mortality of A. aegypti larvae after exposure to infective juveniles of H. indica. [file peerj-09-11633-s001.pdf]
